# Bioremediation of Contaminated Soil by Fungi: A Call for Research

**DOI:** 10.3390/jof10100684

**Published:** 2024-09-29

**Authors:** Fayuan Wang, Linchuan Fang, Zhaoyong Shi

**Affiliations:** 1 College of Environment and Safety Engineering, Qingdao University of Science and Technology, Qingdao 266042, China; 2 School of Resources and Environmental Engineering, Wuhan University of Technology, Wuhan 430070, China; 3 College of Agriculture, Henan University of Science and Technology, Luoyang 471003, China

Soil contamination represents a global environmental challenge, posing a threat to soil ecosystems, agricultural production, and human health. Bioremediation using microorganisms, plants, or soil fauna is considered a low-cost and environmentally friendly soil remediation technology. As one of the most diverse groups of organisms on Earth, fungi contribute greatly to maintaining multiple ecosystem functions and services, particularly litter decomposition, nutrient cycling, disease and pest control, and pollutant degradation and detoxification [1,2]. Abundant evidence has confirmed that numerous fungi are able to degrade or transform organic chemicals, toxic metal(loid)s, and radionuclides through various detoxification mechanisms, showing great potential for the bioremediation of contaminated soils [3,4]. In addition, symbiotic fungi such as mycorrhizal fungi can help host plants to survive in stressful environments through improving their tolerance to contaminants, thus facilitating phytoremediation of contaminated sites [1]. However, due to the complexity of soil contaminants, there are still large knowledge gaps to be bridged in the mycoremediation of contaminated soil. Increasing concerns regarding soil contamination and remediation technologies call for new insights into soil bioremediation using fungi.

In this Special Issue, two reviews and three original research papers were published, focusing on the latest advances on soil mycoremediation. A series of soil pollutants were involved, including heavy metals like cadmium (Cd) and molybdenum (Mo), organic contaminants from crude oil, and diverse contaminants caused by war-like activities (e.g., explosives, metal(loid)s, radionuclides, and herbicides). The main findings of these studies are briefly summarized below.

Arbuscular mycorrhizal (AM) fungi are geographically ubiquitous in terrestrial ecosystems that can form mutualistic symbiosis with the vast majority of vascular plants. Previous studies have confirmed that AM fungi can contribute to the detoxification of various toxic metal(loid)s and the maintenance of soil and plant health [1,5]. An appropriate amount of Mo is beneficial for plants and animals; however, it is toxic in excess. Human activities such as mining and smelting can cause soil Mo contamination. Zhang et al. [6] explored the effect of different AM fungi on maize plants exposed to different levels of Mo stress. They found that both *Claroideoglomus etunicatum* and *Rhizophagus intraradices* promoted plant growth and alleviated the Mo-induced phytotoxicity by regulating the allocation of Mo in plant tissues and improving the leaf photosynthesis performance and the plant uptake of nutrients. Different from Mo, Cd is among the most toxic metals and the most common metallic pollutant in the agricultural soils of China. Lao et al. [7] found that inoculation with *Funneliformis mosseae* reduced the transfer of Cd from maize roots to shoots, which was achieved by enhanced Cd compartmentalization induced by increased lignin in the cell walls. The authors also found that AM colonization activated the activity of enzymes involved in lignin synthesis and increased Cd-binding functional groups of lignin. These results provide valuable insights into AM fungi-assisted phytoremediation of soil polluted with Mo and Cd.

However, the efficiency of AM fungi-assisted phytoremediation depends on the toxicity and concentration of the contaminants, the plant type and tolerance, and the soil conditions. AM fungi cannot grow and colonize normally in unplanted soil and severely polluted soil. Soil amendments such as biochar have been proposed to immobilize metallic contaminants, regardless of the pollution degree, which may help to protect AM fungi and interact synergistically with them to facilitate soil remediation. Fang et al. [8] critically reviewed the existing research on soil Cd immobilization by the joint action of biochar and AM fungi. Concretely, they can collaborate in decreasing the availability and mobility of Cd, improving plant nutrition, and reducing Cd toxicity and plant Cd accumulation. Many soil amendments have been developed to immobilize potentially toxic elements, some of which may benefit mycoremediation. This review helps to explain the combined remediation strategy for soil contamination.

Crude oil and its toxic substances have been identified as prevalent soil contaminants with great environmental and health risks. Gaid et al. [9] isolated eight filamentous fungal strains from various soil samples in Kazakhstan and tested their biodegradation capacities. The results showed that *Aspergillus* sp. SBUG-M1743 and *Penicillium javanicum* SBUG-M1744 exhibited remarkable biodegradation capabilities for tetradecane, whereas *Fusarium oxysporum* SBUG-M1746 displayed a good ability to degrade cyclohexanone and cyclohexane. These findings mean that fungal species and strains in various habitats may have developed different biodegradation capacities. Considering the species and functional diversity of soil fungi, mycoremediation represents a feasible strategy for the remediation of oil-polluted soil.

In addition to heavy metals and crude oil, soil contaminants caused by war-like activities are also addressed in this Special Issue [10]. Notably, war-like activities generally cause the soil’s combined pollution, including explosives, metal(loid)s, radionuclides, and herbicides. Using a comprehensive review, Geris et al. [10] summarized the biosorption, bioaccumulation, biodegradation, and biotransformation of these pollutants by various fungi and introduced a number of successful cases in the mycoremediation of soil polluted through war-like activities. However, most previous studies have focused on explosives. There are still challenges for the applications of mycoremediation for soils contaminated by multiple pollutants, particularly persistent organic pollutants with a low degradability.

We would like to thank all the authors and reviewers for their contributions. It was their efforts that ensured the successful release of this Special Issue, which included five articles. Due to the complexity and heterogeneity of soil systems, there are still large challenges for the development and optimization of effective soil remediation technologies. Fungal remediation provides an option; however, it is not omnipotent and requires further improvement. We call for more efforts from the global scientific community.
